# Upconversion nanoscopy revealing surface heterogeneity of tumor-secreted extracellular vesicles

**DOI:** 10.1038/s41377-023-01106-5

**Published:** 2023-03-03

**Authors:** Rui Pu, Qiuqiang Zhan

**Affiliations:** Centre for Optical and Electromagnetic Research, South China Academy of Advanced Optoelectronics, South China Normal University, Guangzhou, 510006 China

**Keywords:** Super-resolution microscopy, Nanoparticles

## Abstract

Super-resolution microscopic imaging employing upconversion nanoparticles is applied to reveal the surface heterogeneity of tumor cell-derived small extracellular vesicles, i.e., exosome. The number of surface antigens of every extracellular vesicles can be quantified by both the high imaging resolution and stable brightness of upconversion nanoparticles. This method proves its great potential in nanoscale biological studies.

Extracellular vesicles (EVs) are cell-derived micro-/nanoparticles enclosed by a lipid bilayer and contain biological cargos including lipids, proteins, DNA, and RNA. As mediators of intracellular communications, EVs not only play an important role in regulating fundamental biological processes, but also involved in disease pathogenesis, such as the tumour progression or the spread of numerous pathogens^1^. Observing the structure of EVs has always been a major need for understanding the action mechanism, but the small sizes of EVs (e.g., exosomes with a diameter of 40–120 nm) make them only being characterised by a few techniques. The non-invasive and bio-friendly optical nanoscopy (super-resolution microscopy) is an ideal tool for this purpose, but either the commonly used single-molecule localisation microscopy (SMLM) or stimulated emission depletion microscopy (STED) suffers from problems such as low throughput, high power requirement, or failure to support long-period observation. In a recent article published in eLight^2^, Huang and co-workers proposed an ingenious strategy to reveal the surface heterogeneity of tumor-secreted EVs, by employing super-resolution microscopy based on lanthanide-doped upconversion nanoparticles (UCNPs). Previously, this group has already demonstrated the feasibility and advantages of using UCNPs for quantitative EVs detection^3^.

UCNPs are an emerging inorganic luminescence probe with unique nonlinear excitation property of stepwise upconversion. The nonlinear excitation process, including multiphoton excitation and saturated excitation, has been cleverly applied to super-resolution imaging, which gives rise to many novel techniques with distinct advantages^4–6^. Another main reason driving people to upconversion nanoscopy is the high photostability, which supports long-period imaging without any photobleaching or blinking^7–9^. With surface functionalization and immunolabelling techniques, these inorganic nanoparticles can be targeted to specific biological structures or molecules for bioimaging and detection. In Huang et al.’s work, tumour epitope epithelial cellular adhesion molecule (EpCAM) on the surface of EVs was selected for study. The EVs were captured and fixed on an antibody-coated sample plate, and the UCNPs were targeted to EpCAM with biotin-avidin labelling method (Fig. 1). The researchers used a laser-scanning microscopic imaging system with a doughnut-shaped excitation beam for upconversion super-resolution imaging. Here, with the nonlinear saturated excitation^4^, the UCNPs would be imaged as doughnut-shaped spots with a new profile which differs from that of the commonly-used Gaussian beam. The higher the degree of saturated excitation, the smaller the area of the central hole of the spot, and thereby the imaging provides a higher resolution.

**Fig. 1:**
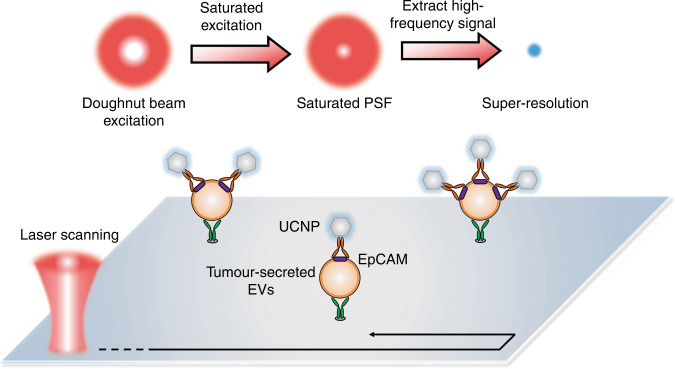
Upconversion nanoscopy for EVs surface molecules quantification. By using saturated excitation of doughnut-shaped beam, both the high-resolution images and quantifiable luminescence intensity can reveal the number of labelled upconversion nanoparticles on each vesicle

The specific labelling of UCNPs was first demonstrated by comparing with the genetically encoded green fluorescent protein (mGFP), which expressed together with EpCAM. Pearson’s R-value of 0.83 for large vesicles and ~65% co-localisation degree for small vesicles were demonstrated in co-localisation experiments. The researchers then used the super-resolution imaging system to count the number of UCNPs labelled on every EV, which can theoretically reflect the quantity distribution of EpCAM molecules. It should be noted that the UCNPs with uniform brightness can not only provide 40-nm resolution images for directly observation and counting, the brightness of clusters can also be used to distinguish the number of nanoparticles. The researchers found that by using 40-nm UCNPs as luminescence probes, most vesicles were labelled by 0 to 3 nanoparticles, but when the size of UCNPs decrease to 27 nm and 18 nm, the maximum labelling number would increase to 9 and 21. The results show that EVs with more labelled UCNPs have a less probability, which to some extent reflect the heterogeneity of EpCAM molecules. Moreover, the number distribution of UCNPs labelled on EVs is inherently of great research value. The changes in distribution may reflect different progression conditions of tumour, but this needs to be investigated in future studies.

In our opinion, the method proposed by Huang and co-workers has at least three distinct merits. First, the advantages of upconversion super-resolution imaging, such as bio-friendly near-infrared excitation, high signal-to-background ratio, and high photostability, are wisely exploited in their biological detection method, which provides significantly better detection accuracy than the commonly used organic dyes. Second, the demonstrated upconversion super-resolution microscopy with high spatial resolution and unfading brightness to quantify the biomolecules of target structures is a smart approach with great research and practical value. Apart from the direct application for clinical monitoring the cancer progression or treatment, this method may also be applied to other biomolecules or physiology, such as immune surveillance and drug delivery. In addition, this powerful method itself is an inspiring biological application example for the study of UCNPs. Therefore, it will not only benefit the bioimaging and biomedical fields, but also drive the further development of upconversion nanotechnology.

## References

[1] El Andaloussi S (2013). Extracellular vesicles: biology and emerging therapeutic opportunities. Nat. Rev. Drug Discov..

[2] Huang G (2022). Upconversion nanoparticles for super-resolution quantification of single small extracellular vesicles. eLight.

[3] Huang G (2022). Single small extracellular vesicle (sEV) quantification by upconversion nanoparticles. Nano Lett..

[4] Chen CH (2018). Multi-photon near-infrared emission saturation nanoscopy using upconversion nanoparticles. Nat. Commun..

[5] Liang YS (2022). Migrating photon avalanche in different emitters at the nanoscale enables 46th-order optical nonlinearity. Nat. Nanotechnol..

[6] Wu QS (2017). Non-bleaching fluorescence emission difference microscopy using single 808-nm laser excited red upconversion emission. Opt. Express.

[7] Liu YJ (2017). Amplified stimulated emission in upconversion nanoparticles for super-resolution nanoscopy. Nature.

[8] Zhan QQ (2017). Achieving high-efficiency emission depletion nanoscopy by employing cross relaxation in upconversion nanoparticles. Nat. Commun..

[9] Pu R (2022). Super-resolution microscopy enabled by high-efficiency surface-migration emission depletion. Nat. Commun..

